# Direct modulation index: A measure of phase amplitude coupling for neurophysiology data

**DOI:** 10.1002/hbm.26190

**Published:** 2022-12-29

**Authors:** Maximilian Scherer, Tianlu Wang, Robert Guggenberger, Luka Milosevic, Alireza Gharabaghi

**Affiliations:** ^1^ Institute for Neuromodulation and Neurotechnology University Hospital and University of Tübingen Tübingen Germany; ^2^ Krembil Brain Institute University Health Network Toronto Canada; ^3^ Institute for Biomedical Engineering University of Toronto Toronto Canada

**Keywords:** connectivity, cross‐frequency coupling, neural oscillations, phase‐amplitude coupling

## Abstract

Neural communication across different spatial and temporal scales is a topic of great interest in clinical and basic science. Phase‐amplitude coupling (PAC) has attracted particular interest due to its functional role in a wide range of cognitive and motor functions. Here, we introduce a novel measure termed the direct modulation index (dMI). Based on the classical modulation index, dMI provides an estimate of PAC that is (1) bound to an absolute interval between 0 and +1, (2) resistant against noise, and (3) reliable even for small amounts of data. To highlight the properties of this newly‐proposed measure, we evaluated dMI by comparing it to the classical modulation index, mean vector length, and phase‐locking value using simulated data. We ascertained that dMI provides a more accurate estimate of PAC than the existing methods and that is resilient to varying noise levels and signal lengths. As such, dMI permits a reliable investigation of PAC, which may reveal insights crucial to our understanding of functional brain architecture in key contexts such as behaviour and cognition. A Python toolbox that implements dMI and other measures of PAC is freely available at https://github.com/neurophysiological-analysis/FiNN.

## Graphical abstract

Neural coupling measures are sensitive to higher harmonics of the target oscillation. The direct modulation index is a novel measure that achieves frequency‐specificity by sine‐fitting the phase‐amplitude histogram. dMI allows for reliable estimation of phase‐amplitude coupling with increased robustness to noise and signal duration in comparison to other measures.

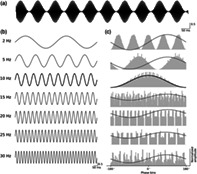

**Abbreviations**
dMI: direct modulation index
EEG: electroencephalography
MI: modulation index
MVL: mean vector length
PAC: phase‐amplitude coupling
PLV: phase‐locking value

## 1 INTRODUCTION

The investigation of the communication between structures across different spatial and temporal scales has been a major area of interest in the field of cognitive and motor neuroscience (Siems et al., 2016; Siems & Siegel, 2020). In particular, a growing body of research regards phase‐amplitude coupling (PAC) as a phenomenon reflective of a multi‐frequency communication mode across and within neural structures (Canolty & Knight, 2010; Jensen & Colgin, 2007). The level of PAC between neural structures is quantified by the degree to which the phase of a low‐frequency neural oscillation reflects the shape of the amplitude of a high‐frequency oscillation (Bragin et al., 1995; Lakatos et al., 2005). Several studies have reported a close relationship between PAC of high‐gamma amplitude with alpha phase and behavioral performance on cognitive, motor, and sensory tasks (e.g., Schroeder & Lakatos, 2009; Voytek et al., 2010; Yanagisawa et al., 2012).

Given the large interest in PAC, many methods have been developed to provide an accurate quantification of this cross‐frequency neural communication (Tort et al., 2010). A non‐exhaustive overview of established methods is presented in Table 1. The methods include the modulation index (MI; Tort et al., 2008), mean vector length (MVL; Canolty et al., 2006) and phase locking value (PLV; Mormann et al., 2005). Although these methods were successful in revealing relevant brain‐behaviour relationships (e.g., Canolty & Knight, 2010; Penny et al., 2008), they share two main limitations. First, none of these methods provides a bounded output measure, which prohibits the interpretation of absolute PAC values across different studies (Hülsemann et al., 2019; Tort et al., 2010). Second, some methods may erroneously detect high PAC values at harmonic multiples of the frequency of the enveloped amplitude signal (e.g., Giehl et al., 2021; Kramer et al., 2008), as suggested by the findings of the present investigation.

**TABLE 1:** Overview of phase‐amplitude coupling metrics

Measure	Bounded output	False positives at higher harmonics	Performs well at lower SNR	Performs well with shorter signal durations
Phase‐locking value (Mormann et al., 2005)	No	Yes	No	Yes
Mean vector length (Canolty et al., 2006)	No	Yes	No	No
Modulation index (Tort et al., 2008)	No	No	No	Yes
Direct modulation index	Yes	No	Yes	Yes

This work introduces the direct modulation index (dMI) as a novel measure of PAC, which aims to circumvent the limitations of the aforementioned methods. The dMI is a bound variation of the MI as introduced by Tort et al. (2008). A dMI value of 1 indicates strong PAC, while a value of 0 indicates no PAC. Furthermore, dMI is highly sensitive to the target frequency only, and therefore avoids the pitfall of assuming significant PAC changes at harmonic frequencies to be actual findings. In the next section, we begin with a description of the proposed measure, followed by an illustration of its performance in comparison to a selection of established connectivity methods on simulated data.

## 2 METHODS

### 2.1 Direct modulation index

With the modulation index as its first step, the dMI shares the calculation of a phase‐amplitude histogram (Tort et al., 2008). Following preprocessing, which includes bandpass filtering; a phase‐amplitude histogram is constructed across the entire duration of the input signal by extracting the phase of the low‐frequency signal and the amplitude of the high‐frequency signal. In the current implementation, the Hilbert transform was used to extract the phase of the low‐frequency signal, while the amplitude of the high‐frequency signal was estimated using the rectified signal rather than the Hilbert‐transform in order to speed up processing. The original study by Tort et al. constructed the phase‐amplitude histogram using 18 phase bins of 20° wide. Conversely, we opted to use 360 overlapping phase bins of 20° width each, shifted in steps of 1°, in order to obtain a better model fit in the following step. Nevertheless, comparisons between the dMI to the MI, both calculated with the original 18 bins of 20° each have been added in Supplementary Materials 1. A composite signal is then constructed from the phase of the low‐frequency signal and the amplitude of the high‐frequency signal, and the mean amplitude is calculated across phase bins. In the next step, the phase‐amplitude histogram is normalized to produce a more robust fit in the following step. To ensure that it is more resistant towards outliers, the 25^th^ and 75^th^ percentiles–as opposed to the minimum and maximum values–are chosen as reference points for the normalization. The values are selected based on the interquartile range, and have been shown to be robust against outliers when the proportion of outliers is less than 25% (Jones, 2019). Next, the values are scaled and shifted to result in a normalized histogram which is loosely bound to the interval [−1, 1]. Afterwards, instead of scoring the PAC values from entropy, a sinusoid is fitted to the normalized histogram. We used a non‐linear least‐squares algorithm, implemented in the LMFIT package in Python (Newville et al., 2015). The frequency of the sine is set to 1 cycle (per 360°), while the phase and amplitude are preset to 0 and 1, respectively. During the fit, the phase is allowed to vary between −180 and +180°, and the amplitude is allowed to vary between 0.95 and 1.05 in order to obtain the best fit. We selected a sinusoidal function because the phase‐amplitude histogram of two signals with an ideal PAC relationship was observed to default to a sinusoid shape. Fitting a sinusoid through the phase‐amplitude histogram renders the measure highly sensitive to the targeted frequency only, whereas the entropy metric is also sensitive to the harmonic frequencies. Finally, an error value is calculated by taking the squared difference between the height of each individual phase bin and the amplitude of the sinusoidal fit at the corresponding bin. The errors are averaged across phase bins, capped at 1, and then subtracted from 1 to arrive at the dMI. This final step sets the lower and upper bounds of the dMI to 0 and 1, respectively. It simplifies the interpretation of the PAC estimate, as values approaching zero indicate low‐coupling strength, while values approaching 1 indicate strong coupling.

### 2.2 Validation data

**FIGURE 1:**
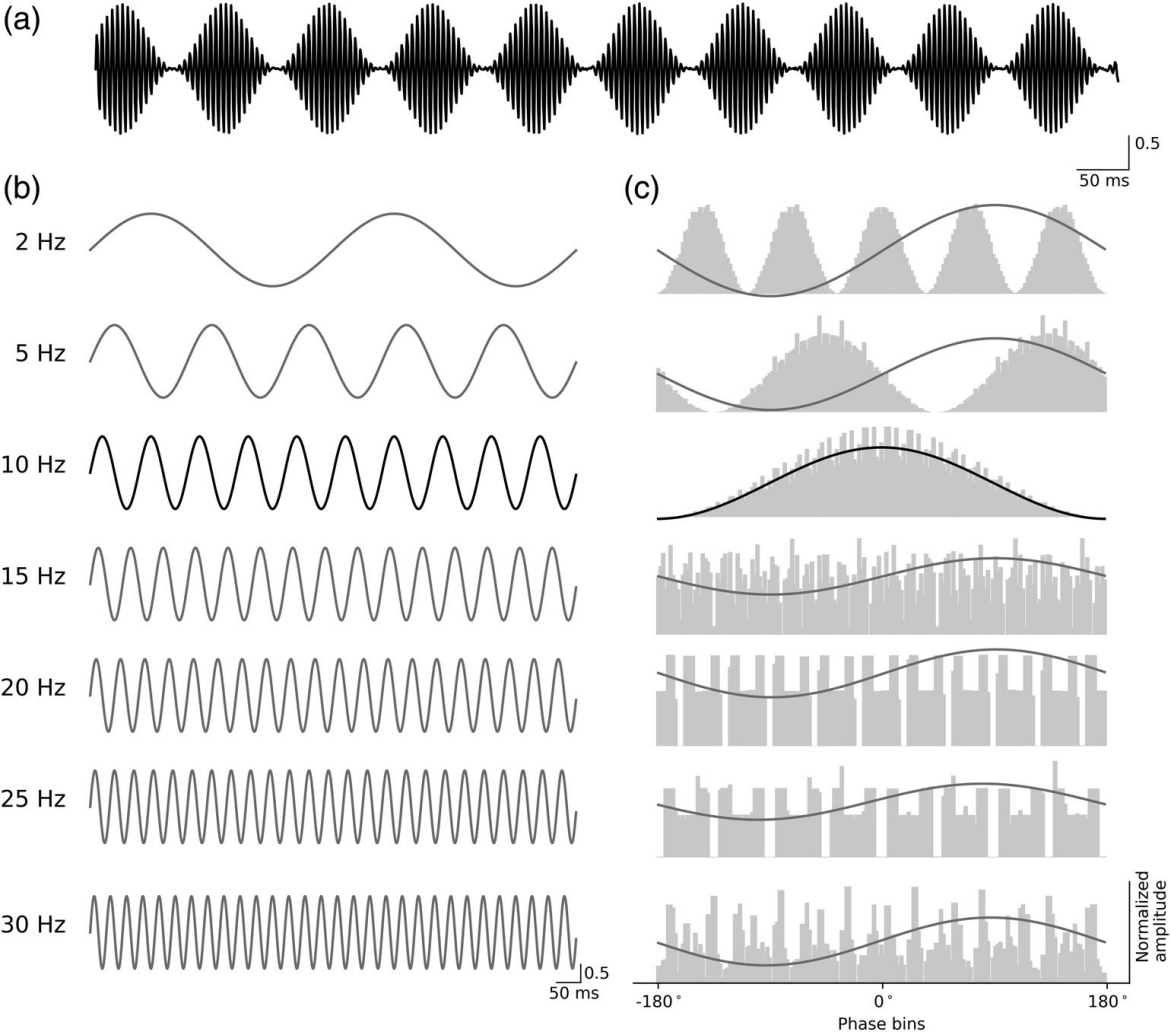
Simulated signals and calculation of direct modulation index (dMI). (a) High‐frequency signal with an amplitude (in arbitrary units) that was modulated by a 10 Hz low‐frequency signal. (b) Low‐frequency signals used in the validation of dMI. The 10 Hz signal in the third row represents the low‐frequency signal that modulates the high‐frequency signal in (a). (c) Histograms of the amplitude of the high‐frequency signal from (a) binned according to the phase of the low‐frequency signals in (b) are shown in grey. The y‐axis shows the mean amplitude in arbitrary units, the x‐axis shows the phase bins in normalized units. The best sine fit is plotted over the histogram. The goodness of fit is calculated from the difference between the fitted sine and the height of the individual histogram bins. In the event of a good fit, as with the 10 Hz low‐frequency signal in the third row, the sine fit lines up with the phase‐amplitude histogram

dMI was evaluated in comparison to the following PAC methods: MI (Tort et al., 2008, 2010), MVL (Canolty et al., 2006), and PLV (Mormann et al., 2005). The reader may refer to the corresponding articles for a description of the evaluated measures. dMI and the aforementioned PAC methods were evaluated using simulated data. As opposed to using experimental data–where it is unclear whether any detected PAC at harmonic frequencies is reflective of a true coupling–simulated data enabled us to absolutely determine any signal properties, including the confirmable absence of PAC at the higher harmonic frequencies. We generated a high frequency signal of 200 Hz with an amplitude that was modulated by a 10 Hz oscillation (Figure 1a), and several low frequency signals of 2, 5, 10, 15, 20, 25, and 30 Hz (Figure 1b). Each signal had a duration of 30 s and a sampling rate of 1000 Hz. A high PAC value was anticipated for the 10 Hz low frequency signal, but not for the other low frequency signals (Figure 1c). PAC was modeled to be consistenly present throughout the signal without fluctuations in strength.

To test the performance of each PAC method at different signal‐to‐noise ratios, Gaussian noise, with amplitudes that are 0%, 25%, 50%, 100%, or 150% of the amplitude of the signal, was introduced. To additionally investigate the combined effects of noise level and signal duration on each PAC method, the analysis was repeated with a signal length of 300 s. Investigations of the effect of noise with additional signal durations of 0.5 s and 1 s have been included in Supplementary Materials 2. Finally, the performance of each method with varying signal durations was tested by capping the signals at durations of 500, 600, 700, 800, 900, and 1000 ms. In this evaluation, the amplitude of the Gaussian noise was fixed at 33% of the signal amplitude.

## 3 RESULTS

**FIGURE 2:**
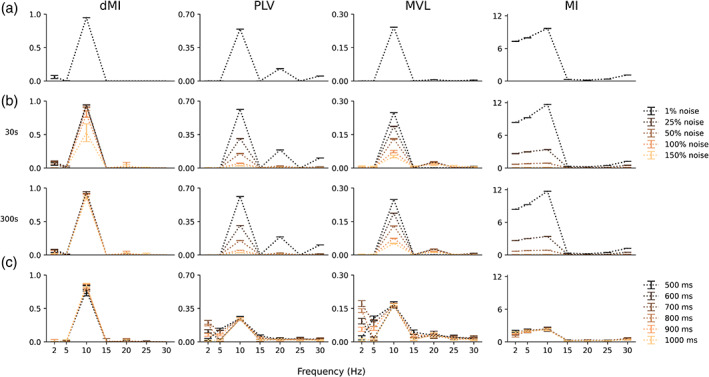
Comparison of PAC measures in simulated data. The columns show the performance of direct modulation index (dMI), phase‐locking value (PLV), mean vector length (MVL), and the classical modulation index (MI). The y‐axes show the PAC estimates in arbitrary units (mean and standard deviation). (a) PAC estimates on simulated signals of 30 s and 5% Gaussian noise. (b) PAC estimates on simulated signals of 30 s (top row) and 300 s (bottom row) with varying levels of Gaussian noise. (c) PAC estimates on simulated signals of varying durations and 33% Gaussian noise

Figure 2 shows the PAC estimates by dMI, MI, MVL, and PLV at various levels of SNR and signal durations. A summary of the performance of the measures can be found in Table 1.

Figure 2a shows the PAC estimates obtained using dMI, MI, MVL, and PLV. A consistent, single peak at 10 Hz can be observed for dMI, with a PAC value of 0.94. Furthermore, an elevated dMI value of 0.064 can be observed at 2 Hz. PAC estimates from MVL showed a prominent peak at 10 Hz, with a value of 0.24. Furthermore, elevated MVL values are also observed at 5 and 25 Hz, with values of 0.001 and 0.006, respectively. PLV‐based estimates showed the largest peak at 10 Hz with a PAC value of 0.54. However, at frequencies of 20 and 30 Hz, prominent peaks of 0.13 and 0.06, respectively, also appeared. Finally, MI‐based estimates showed the highest PAC values of 9.70 at 10 Hz, as well as elevated values of 7.30 and 7.96 at the lower frequencies of 2 Hz and 5 Hz, respectively.

Figure 2b shows the effect of varying levels of Gaussian noise on the PAC estimates of the investigated measures for signal lengths of 30 s and 300 s. A general reduction in PAC values can be observed for increasing noise levels. PAC estimates based on MI and PLV decreased most (61% and 51%, respectively) in the presence of weak noise (25% of the signal amplitude). By comparison, the MVL‐based estimates decreased by 25%, and the dMI‐based estimates decreased by 0.29% only. Also, across different Gaussian noise levels, dMI drop rates were consistently lower than those of the other evaluated measures. Furthermore, increasing the signal length to 300 s increased the robustness against noise for the dMI‐based PAC estimates, but not for the other measures (Figure 1b, bottom row). Finally, the erroneously elevated PAC values of MI and PLV were also present in the analysis across different levels of Gaussian noise.

Figure 2c shows the effect of signal length on the PAC estimates of the measures investigated. The results were similar to those reported in the previous investigation of the effect of noise. Decreasing the signal length did not have an effect on dMI‐based PAC estimates. When using the MI to estimate PAC, small fluctuations were observed as an effect of signal length. MVL and PLV appeared to be most affected by signal length, especially for the low frequency signals of 2 Hz and 5 Hz. The erroneously elevated PAC values of MI and PLV were also present in the analysis across different signal lengths.

## 4 DISCUSSION

The current work presents the dMI as a novel measure of PAC. Our dMI has been designed to be easily interpretable on a stationary interval between 0 and +1, and specific to the frequency of interest only. The performance of dMI, PLV, MVL, and MI was investigated using artificial data under increasing levels of noise and with decreasing amounts of data. The results indicate that dMI is more robust towards varying levels of Gaussian noise and short signal durations than the other PAC methods investigated in the scope of our evaluations (Table 1). The dMI measure, as well as other reliable measures to estimate neurophysiological interactions for example, in the same frequency band (Scherer et al., 2022a), is freely available as a Python toolbox at https://github.com/neurophysiological-analysis/FiNN (Scherer et al., 2022b).

First, one characteristic specific to dMI is that its PAC estimates are bound to a stationary interval between 0 and 1, as opposed to the other investigated methods where the estimates can theoretically take on a wide range of values. Bounding the output to a specific, stationary interval allows for the interpretation of absolute changes in PAC. This, in turn, facilitates the calculation of meaningful effect sizes in statistical investigations. A combination of these two pieces of information is essential for any meaningful interpretation and for the discussion of any results (Lakens, 2013; Stankovski et al., 2017).

Second, dMI was found to be highly resilient towards high levels of Gaussian noise and performed well with decreasing amounts of data within the extent of the current investigations. By contrast, PAC estimates from MI, PLV, and MVL strongly deteriorated as the levels of noise increased. While the decreasing signal duration had no effect on dMI, for PLV it led to a high number of erroneously elevated PAC estimates at lower frequencies.

Finally, in our investigations, we observed that MI tends to systematically overestimate PAC values for frequencies below the target frequency. This may be related to the scoring mechanism of MI, which calculates the entropy of the phase‐amplitude histogram. This entropy was still high when low levels of Gaussian noise were added. MVL‐based PAC estimates also showed poor performance at frequencies lower than the target frequency, in particular for shorter signal lengths, as it returned a high number of erroneously elevated PAC estimates within that range. The dMI, while minimal and little affected by noise or signal length, slightly overestimated PAC at 2 Hz. This may be related to the fit of the sinusoid to the phase‐amplitude histogram, which approaches the histogram as the frequency decreases. On the other hand, both PLV and MVL tended to systematically overestimate PAC values at the harmonics of the target frequency. These observations underline the sensitivity of PAC estimates, and the need for further investigation towards a reliable solution. However, it is important to bear in mind that these observations are potentially biased, as the results are derived from artificially created data.

The current implementation of dMI assumes a sinusoidal distribution of amplitudes across the individual phase bins. This assumption is likely to hold, provided a sufficiently large sample size with independent measurements is available (Nixon et al., 2010). Our implementation of dMI also enables the user to easily visualize the phase‐amplitude histogram to understand the shape of the PAC fit. In the event that the observed histogram is not Gaussian, the user may conveniently define another function for the shape for the line‐fitting.

## 5 CONCLUSIONS

Here, we presented dMI as a new measure to estimate PAC of neurophysiological data on the basis of a sinusoidal fit of the phase‐amplitude histogram between two signals. The dMI has been designed to be resistant against varying levels of noise, to perform well with short signal durations, and to be easily interpretable due to the absolute boundary values of 0 and +1. Furthermore, through configurations of the parameters and/or changing of the sinusoidal scoring function, dMI is easily adaptable to the question at hand. We used simulated data to show that dMI provides a more reliable estimate of PAC than a number of other established measures. This novel measure may therefore provide a useful tool for the investigation of brain dynamics with implications for basic and clinical science. Future studies are required to test the performance of dMI in real‐life signal processing scenarios in comparison to the other PAC metrics.

### AUTHOR CONTRIBUTION

Maximilian Scherer: Conceptualization, Methodology, Software, Writing–original draft, Writing–review & editing.

Tianlu Wang: Writing–original draft, Writing–review & editing.

Robert Guggenberger: Conceptualization, Methodology, Writing–review & editing.

Luka Milosevic: Conceptualization, Methodology, Writing–review & editing.

Alireza Gharabaghi: Conceptualization, Writing–review & editing, Funding acquisition.

## CONFLICT OF INTEREST

The authors declare no conflict of interest.

## Supporting information


**Data S1:** Supporting Information

## Data Availability

The code of this analysis is available at https://github.com/VoodooCode14/dmi. The implementation of dMI is available at https://github.com/neurophysiological-analysis/FiNN.
