# Supplementary material for: Direct modulation index: A measure of phase amplitude coupling for neurophysiology data
Source: Hum Brain Mapp. 2022 Dec 29;44(5):1862–7. doi: 10.1002/hbm.26190 (PMC9980882; doi:10.1002/hbm.26190)
Supplement: Supplementary file 1 — Data S1: Supporting Information [file HBM-44-1862-s001.docx]

# *Supplementary Materials*

# Direct Modulation Index: a measure of phase amplitude coupling for neurophysiology data

Maximilian Scherer^a,^[[1]](#footnote-2)^^, Tianlu Wang^a^, Robert Guggenberger^a^, Luka Milosevic^a,1^, Alireza Gharabaghi^a§^

^a^Institute for Neuromodulation and Neurotechnology, University Hospital and University of Tübingen, Tübingen, Germany

^§^Corresponding author: Professor Alireza Gharabaghi. Address: Institute for Neuromodulation and Neurotechnology, University Hospital Tübingen, Otfried-Müller-Str. 45, 72076 Tübingen, Germany. Email: alireza.gharabaghi@uni-tuebingen.de

**ORCID**:

Maximilian Scherer: 0000-0002-5280-2828

Tianlu Wang: 0000-0002-7480-9705

Robert Guggenberger: 0000-0003-0970-9705

Luka Milosevic: 0000-0002-4051-5397

Alireza Gharabaghi: 0000-0002-9782-5281

**Running Title**: Direct Modulation Index

**Acknowledgements**

This work was supported by the German Federal Ministry of Education and Research (BMBF INAUDITAS 13GW0270). We acknowledge support by the Open Access Publishing Fund of the University of Tübingen.

**Supplementary Materials 1:**

Figure S1 shows the estimates by dMI and MI at various levels of SNR and signal durations. Here, dMI is calculated using 18 non-overlapping bins of 20 degrees wide, which are the original settings of MI (Tort et al., 2008).

We observed that each of the measures show the highest value at 10 Hz (Fig. S1a). PAC estimates from dMI and MVL furthermore showed smaller peaks at 20 Hz, while PLV also showed a peak at 30 Hz. MI did not show peaks at the higher harmonics of 10 Hz apart from a slight increase at 30 Hz, but showed elevated values at the lower frequencies of 2 Hz and 5 Hz. With less data points to fit the sinusoid to, the dMI became more sensitive to interferences at the harmonic frequencies. However, the dMI remained highly resilient towards high levels of Gaussian noise and decreasing amounts of data in comparison to the other measures (Fig. S1b, S1c).


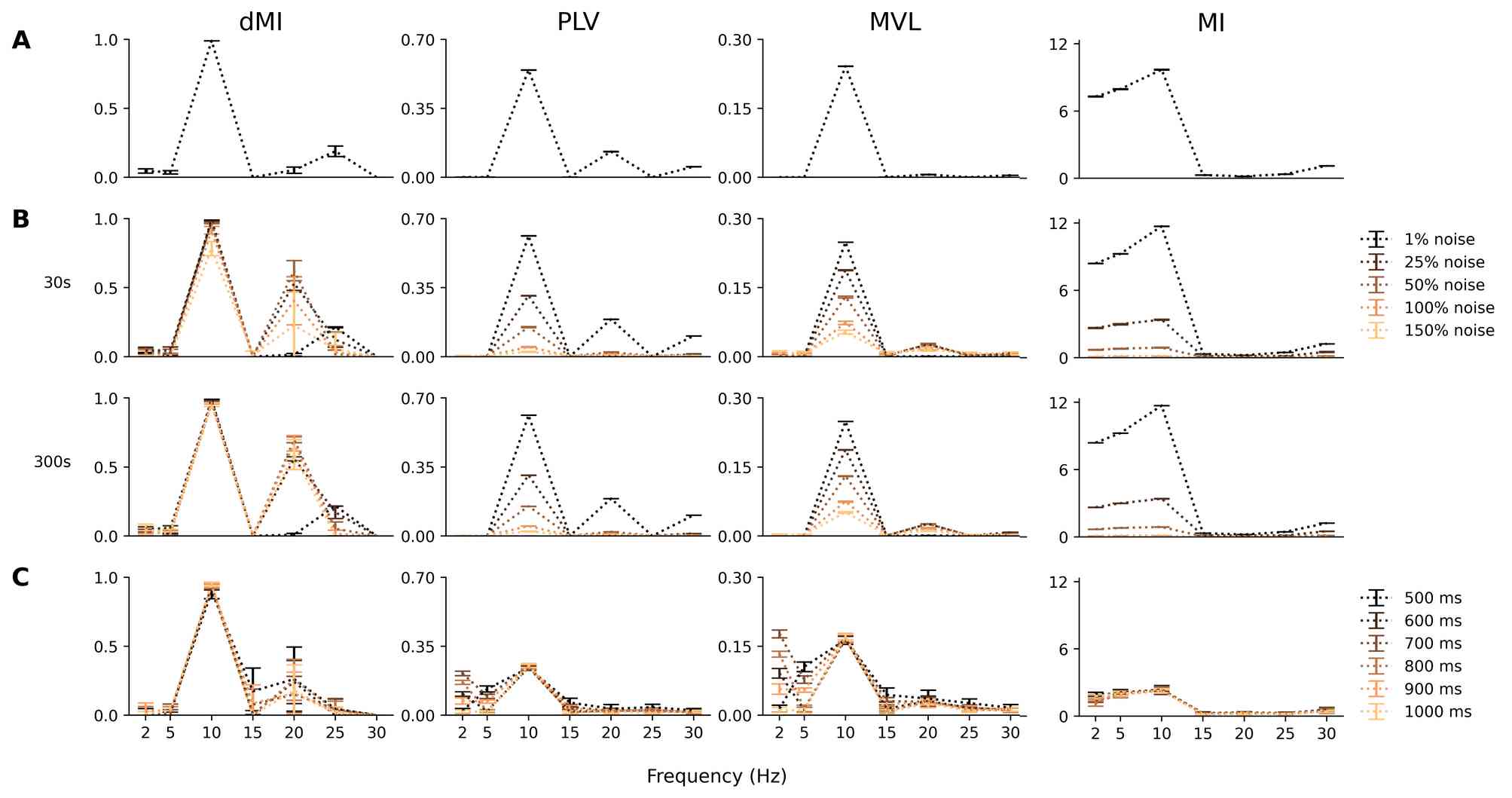
**Figure S1. Comparison of dMI measures calculated with 18 phase bins to other PAC measures in simulated data.** The first column shows the performance of the direct modulation index (dMI) calculated using 18 phase bins with 20 degrees width each. Columns 2, 3 and 4 show the performance of the phase-locking value (PLV), mean vector length (MVL), and the classical modulation index (MI). The y-axes show the PAC estimates in arbitrary units (mean and standard deviation). (**A**) PAC estimates on simulated signals of 30 s and 5% Gaussian noise. (**B**) PAC estimates on simulated signals of 30 s (top row) and 300 s (bottom row) with varying levels of Gaussian noise. (**C**) PAC estimates on simulated signals of varying durations and 33% Gaussian noise.

**Supplementary Materials 2**

Figure S2 shows the effect of varying levels of Gaussian noise on the PAC estimates of the investigated measures for signal durations of 0.5 s, 1 s, 30 s and 300 s. Across all signal durations, a general reduction in PAC values can be observed for increasing noise levels. At the shortest signal durations of 0.5 s and 1 s, dMI only misidentified the peak PAC frequency as 15 Hz and 20 Hz, respectively, at the highest noise level of 150%. PLV and MLV were able to show the highest peak at 10 Hz consistently across all noise levels for a signal of 1 s, however, when signal duration decreased to 0.5 s, the peaks in PLV and MVL shifted to 5 Hz for noise levels above 50% and 25%, respectively. Finally, MI started to show erroneous peaks at 100% noise and a signal duration of 1 s, and at 50% noise and a signal duration of 0.5 s. The results showed that dMI remained the most robust measure against increasing levels of noise at shorter signal durations as well.


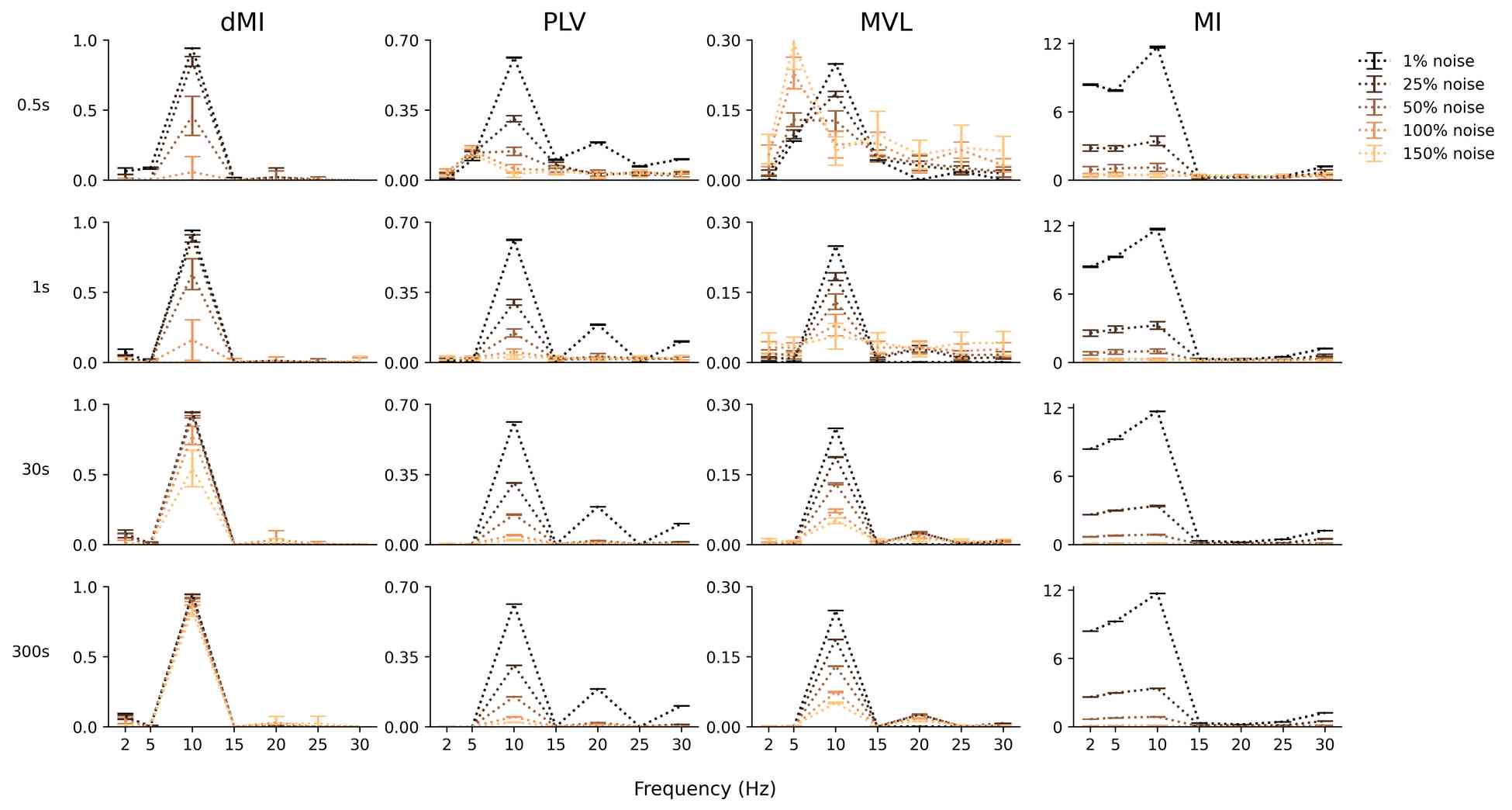
**Figure S2. Comparison of different simulated data segment lengths.** The first column shows the performance of the direct modulation index (dMI) calculated using 360 overlapping phase bins with 20 degrees width each. Columns 2, 3 and 4 show the performance of the phase-locking value (PLV), mean vector length (MVL), and the classical modulation index (MI). The y-axes show the PAC estimates in arbitrary units (mean and standard deviation). The rows show the PAC estimates on simulated signals of 0.5 s, 1 s, 30 s and 300 s with varying levels of Gaussian noise

.

1. Krembil Brain Institute, University Health Network, and Institute of Biomedical Engineering, University of Toronto, Toronto, Canada [↑](#footnote-ref-2)
